# Living alone reduces the decline of calf circumference among Chinese older adults: A 4-year longitudinal study

**DOI:** 10.3389/fragi.2022.1063760

**Published:** 2022-12-15

**Authors:** Dong Wang, Jun Zhang

**Affiliations:** Department of Neurology, Peking University People’s Hospital, Beijing, China

**Keywords:** living alone, calf circumference, older adults, sarcopenia, longitudinal study

## Abstract

**Background:** Calf circumference (CC) is regarded as a surrogate marker of skeletal muscle mass with high sensitivity and specificity for predicting sarcopenia. A cross-sectional study reported older adults living alone were at high risk of developing sarcopenia. Whether living alone affects the change of calf circumference is unknown and there is no evidence from longitudinal study. The purpose of this study was to investigate the relationship between living arrangements and the change of calf circumferences among older adults in China.

**Methods:** The data were from the Chinese Longitudinal Healthy Longevity Survey. A total of 2,203 older adults (age ≥65 years, mean age: 80.61 ± 8.30 years, 50.0% female) who were interviewed in 2014 and then 2018 follow-up survey were finally included for analysis. Living arrangements and other information were collected in 2014. Calf circumferences were measured and recorded in the questionnaires of two waves and the differences were calculated. Logistic regression analyses were conducted to evaluate the association of living arrangements (living alone or not living alone) with the change of calf circumferences (decline or no decline).

**Results:** There were 446 (20.2%) participants living alone and 1,757 (79.8%) participants not living alone. After about 4 years, calf circumferences of 866 (39.3%) older adults declined. Compared to not living alone, living alone was negatively associated with calf circumference decline, even after adjustment for covariates (OR: 0.702, 95% CI: 0.547–0.901, *p* = 0.005). This significant association was found in 65–79 years old (OR: 0.619, 95% CI: 0.407–0.942, *p* = 0.025), female (OR: 0.567, 95% CI: 0.408–0.790, *p* = 0.001) and city/town (OR: 0.461, 95% CI: 0.310–0.685, *p* < 0.001) subgroups but not in advanced age (≥80 years old), male and rural subgroups.

**Conclusion:** This study revealed that living alone was associated with a decreased risk of calf circumference decline among older adults, and might be a protective factor for sarcopenia.

## 1 Introduction

Sarcopenia is defined as age-related loss of skeletal muscle mass, accompanied by decline in muscle strength and/or reduced physical function (Chen et al., 2020). Sarcopenia increases the risks of falls, fractures, disability, and mortality among older adults. Globally, the prevalence of sarcopenia in adults over the age of 60 is 10% (Shafiee et al., 2017), and the prevalence of sarcopenia among older adults over the age of 80 is about 50% (Meng et al., 2014). With an increasingly ageing population, sarcopenia has become a serious public health issue in modern society.

Skeletal muscle mass is one of the core dimensions for assessing sarcopenia. Calf circumference (CC) can be used as a surrogate marker of skeletal muscle mass, which has high sensitivity and specificity for predicting sarcopenia (Kawakami et al., 2015). Measures of CC may be used as a diagnostic proxy in settings where no other muscle mass diagnostic methods are available, and CC < 34 cm for men and <33 cm for women for screening sarcopenia is recommended in the Asian Working Group for Sarcopenia (AWGS) 2019 consensus (Cruz-Jentoft et al., 2019; Chen et al., 2020). In addition, CC is also an important variable in predictive models of other disorders, such as cardiovascular disease (Wu et al., 2018). The CC decline is often regarded as a risk factor affecting health, especially among older adults. Therefore, it is of great significance to explore the factors influencing the change of the CCs among older adults.

Living arrangement is an important observational variable in many older adults-related studies about mental health, cognitive impairment and other diseases (Beghi et al., 2021; Rosenwohl-Mack et al., 2021). Living arrangements are influenced by regional culture and the economic development of different times. Under the influence of Confucianism, the traditional Chinese family is of large size and multigenerational. However, in the new era, the influence of the traditional family concept has declined. Due to the needs of work and the development of urbanization, many young people live far away from their parents, and the traditional family model is gradually deconstructed (Phillips and Feng, 2015). Among older adults, the phenomenon of living alone due to the death of one’s spouses is becoming more and more common. With an increasingly aging population, the older adults living alone has received more and more attention from the society.

The physical and mental health of older adults living alone may be affected when the level of support from family or society changes (Lou and Ng, 2012). It is generally believed that lack of family companionship is harmful to the physical and mental health, but the effects of living alone on the health of older adults are currently inconsistent in different literatures (Holt-Lunstad et al., 2015; Zhou et al., 2018; Gu et al., 2019). Both sarcopenia and frailty are age-related syndromes with some overlap in clinical manifestations (Roberts et al., 2021). Meta-analysis of cross-sectional studies suggested that living alone was a risk factor for frailty in older adults, especially in men (Yamanashi et al., 2015; Kojima et al., 2020); however, meta-analysis of cohort studies did not yield statistically significant results (OR = 0.88, 95 %CI = 0.76–1.03) (Kojima et al., 2020). As for sarcopenia, previous cross-sectional study suggested that older adults living alone are at higher risk for sarcopenia (Cheng et al., 2021), while evidence from cohort studies is still lacking. CC decline among older adults means reduction of skeletal muscle mass and increased risk of developing sarcopenia. There is no report about the associations of some social factors with CC decline among older adults. Therefore, we used the cohort data from the Chinese Longitudinal Healthy Longevity Survey (CLHLS) to explore the relationship between living arrangements and the change of CC among older adults, and analyze the effect of living alone on the changes in skeletal muscle mass and the development of sarcopenia.

## 2 Materials and methods

### 2.1 Study population

**FIGURE 1:**
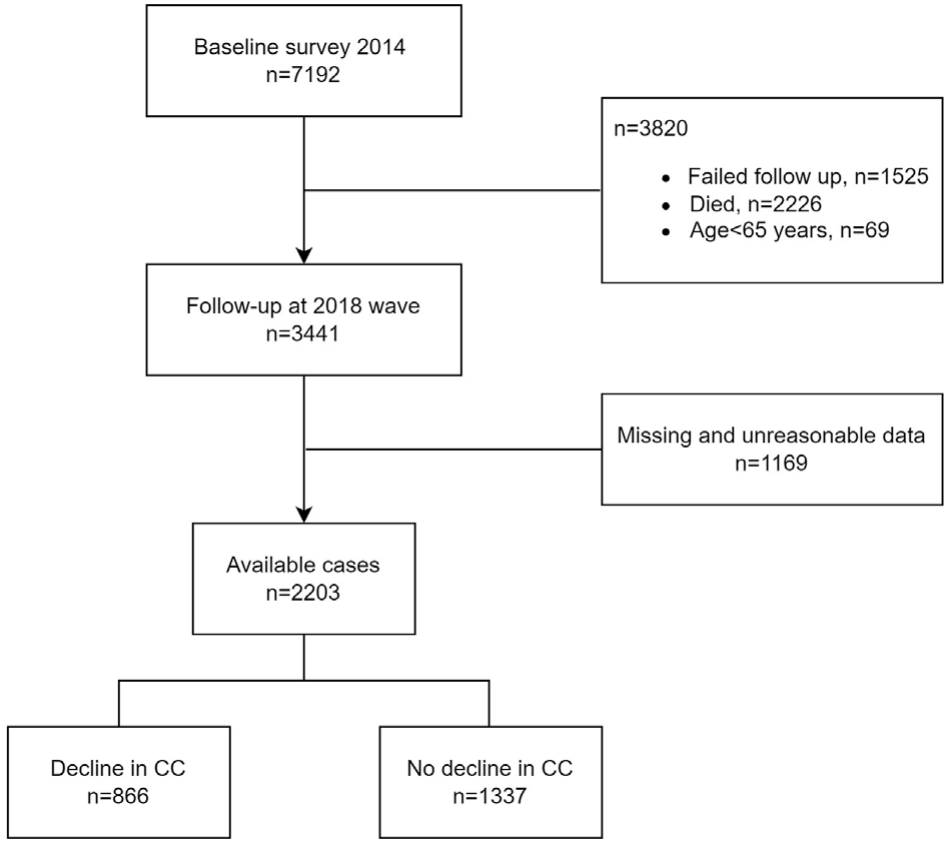
Participant flow in this study.

The data were from CLHLS 2014–2018 longitudinal dataset, which is a nationally representative study of Chinese older adults and covers 23 out of 31 provinces in Mainland China. The CLHLS study was approved by the Research Ethics Committee of Peking University (IRB00001052-13074). The interview was conducted at participants’ homes by well-trained investigators using a structured questionnaire. When a participant was unable to answer questions, the interview was finished by a proxy interviewer, often a spouse or other close relative. At first wave, 7,192 respondents were interviewed in 2014, out of which 1,525 respondents were lost to follow-up at second wave in the 2018 survey and 2,226 died before 2018. Subjects under the age of 65 were excluded. We checked and cleaned the missing data and registration errors of important variables. Finally, 2,203 cases were included for analysis (Figure 1).

### 2.2 Assessment of CC

In this survey, CCs were recorded and rounded up to the nearest integer in centimeters. The CC difference was defined as CC at 2018 wave minus CC at 2014 wave. Given the possible measurement errors and the round-off recording principles, the decline of CC in this survey was defined as CC difference <−1 cm, and CC difference ≥−1 cm means no decline. Finally, there were 866 (39.3%) subjects who had declined CCs.

### 2.3 Living arrangements

Living arrangements were assessed by the item “co-residence of interviewee” in the questionnaire. The options were “with household member(s),” “alone,” and “in an institution,” with 1,735 (78.8%), 446 (20.2%), 22 (1.0%) respondents respectively. The living arrangements were dichotomized as living alone (LA) and not living alone [NLA, including “with household member(s)” and “in an institution”] for analysis.

### 2.4 Covariates

The sociodemographic variables including age, sex, weight (kg), height (cm), education (uneducated or educated), marital status, residence (rural or city/town) and financial support (sufficient or insufficient) were selected from the dataset. The options of current marital status were “currently married and living with spouse,” “married but not living with spouse,” “divorced,” “widowed,” and “never married.” They were dichotomized as “separated/divorced/widowed/single” (SDWS) or not. Body mass index (BMI) was calculated as weight in kilograms divided by the square of height in meters. The sleep quality was categorized as “very good,” “good,” “so,” “bad,” and “very bad” in primitive questionnaire and was dichotomized as bad and not bad for analysis. Sleep duration were recorded in this survey as short (<5 h), medium (5–9 h) and long (>9 h) according to the recommendation (Hirshkowitz et al., 2015). For smoking and drinking, respondents were categorized as current, ex-smoker/drinker, or never. By asking questions: “Exercise or not at present?” and “Have you done physical labor regularly?,” data about exercise/physical labor or not were recorded. Depression was measured using two questions: “Have you felt sad, blue, or depressed for 2 weeks or more in last 12 months?” and “Have you lost interest in most things like hobbies, work, or similar activities?.” Answer of “yes” to any question is considered a representation of depression (Su et al., 2021). Activities of daily living (ADL) disability was defined as needs for support in one or more of the five activities (bathing, dressing, toileting, indoor transferring, and feeding) or being incontinent. A modified Mini Mental State Examination (mMMSE) score was calculated by 23 items in original questionnaire to assess the cognitive function. A detailed introduction to this mMMSE has been published elsewhere (Guo et al., 2022). Every interviewee was asked about diagnoses (hypertension, diabetes, heart disease, stroke, respiratory diseases, tuberculosis, cancer, Parkinson’s disease, arthritis) by hospital.

### 2.5 Statistical analysis

IBM SPSS statistics 26.0 (IBM Corporation, Armonk, NY, United States) was used for statistical analysis. The mean, standard deviation, median, and ratio were calculated for statistical descriptions according to different types of variables. When data was normally distributed, the differences of continuous variables between LA and NLA group at 2014 wave were determined by the independent-sample *t* test, and the homogeneity of variance was tested by Levene’s test. The Mann-Whitney *U* test was used when data was not normally distributed. The statistical analyses of categorical variables were performed by Pearson *χ* ^2^ test. Both univariate and multivariate logistic regression analyses were used to examine the associations of living arrangements with CC difference. Odds ratios (ORs) and 95% confidence intervals (CIs) were calculated. The statistical significance was defined as *p* < .05, and all were two-tailed tests.

## 3 Results

At 2014 wave, there were 446 (20.2%) participants living alone and 1,757 (79.8%) participants not living alone. Table 1 presents the baseline characteristics by two types of living arrangements. No significant differences were found among BMI, sleep quality, exercise, physical labor, depression, ADL disability, and comorbidities. Compared to subjects not living alone, older, women, uneducated, SDWS, rural, insufficient financial support, short-sleeper, non-smoker, non-drinker, and lower mMMSE scorer were more common in subjects living alone.

**TABLE 1:** Baseline characteristics by living arrangements at 2014 wave.

	Living alone (*n* = 446)	Not living alone (*n* = 1,757)	*p*
Age (year)	82.19 ± 7.77	80.21 ± 8.38	**<.001**
Advanced age (≥80 years), *n* (%)	267 (59.9%)	809 (46.0%)	**<.001**
Female, *n* (%)	267 (59.9%)	834 (47.5%)	**<.001**
BMI (kg/m^2^)	22.37 ± 3.81	22.54 ± 3.98	.410
Uneducated, *n* (%)	277 (62.1%)	786 (44.7%)	**<.001**
Marital status (SDWS), *n* (%)	422 (94.6%)	656 (37.3%)	**<.001**
Residence (Rural), *n* (%)	264 (59.2%)	939 (53.4%)	**.029**
Financial support (insufficient), *n* (%)	96 (21.5%)	269 (15.3%)	**.002**
Sleep quality (Bad), *n* (%)	52 (11.7%)	201 (11.4%)	.897
Sleep duration			**.024**
Short, *n* (%)	46 (10.3%)	115 (6.5%)	
Long, *n* (%)	72 (16.1%)	290 (16.5%)	
Medium, *n* (%)	328 (73.5%)	1,352 (76.9%)	
Smoking			**.020**
Current, *n* (%)	78 (17.5%)	349 (19.9%)	
Ex-smoker, *n* (%)	42 (9.4%)	237 (13.5%)	
Never, *n* (%)	326 (73.1%)	1,171 (66.6%)	
Drinking			**.034**
Current, *n* (%)	78 (17.5%)	346 (17.9%)	
Ex-drinker, *n* (%)	33 (7.4%)	191 (10.9%)	
Never, *n* (%)	335 (75.1%)	1,220 (69.4%)	
Exercise, *n* (%)	145 (32.5%)	619 (35.2%)	.281
Physical labor, *n* (%)	382 (85.7%)	1,481 (84.3%)	.478
Depression, *n* (%)	50 (11.2%)	195 (11.1%)	.946
ADL disability, *n* (%)	27 (6.1%)	133 (7.6%)	.271
mMMSE	22.00 (19.00–22.25) ^∗^	22 (20.00–23.00) ^∗^	**.006**
Disease			
Hypertension, *n* (%)	184 (41.3%)	730 (41.5%)	.911
Diabetes, *n* (%)	75 (16.8%)	282 (16.1%)	.695
Heart disease, *n* (%)	98 (22.0%)	348 (19.8%)	.309
Stroke, *n* (%)	67 (15.0%)	257 (14.6%)	.833
Respiratory diseases, *n* (%)	66 (14.8%)	281 (16.0%)	.536
Tuberculosis, *n* (%)	47 (10.5%)	135 (7.7%)	.051
Cancer, *n* (%)	23 (5.2%)	114 (6.5%)	.298
Parkinson’s disease, *n* (%)	22 (4.9%)	92 (5.2%)	.796
Arthritis, *n* (%)	60 (13.5%)	235 (13.4%)	.966

BMI, body mass index; SDWS, separated/divorced/widowed/single; ADL, activities of daily living; mMMSE, modified Mini Mental state examination. *median and 25th–75th percentile.

Bold values represent statistical difference.

The outcomes of logistic regression models are illustrated in Table 2. Compared to NLA group, the OR of the LA group for crude model was 0.728 (95% CI: 0.585–0.905, *p* = .004). After adjustments for covariates, the results were also statistically significant. Living alone was independently and negatively associated with CC decline in adjusted model 2: the adjusted OR of LA group was 0.702 (95% CI: 0.547–0.901, *p* = .005). In adjusted model 2, advanced age (≥80 years) and stroke were two covariates that were significantly associated with CC decline (Table 2).

**TABLE 2:** Associations between CC decline and living arrangements by logistic regression models.

	Crude model		Adjusted model 1		Adjusted model 2	
	OR (95% CI)	*p*	OR (95% CI)	*p*	OR (95% CI)	*p*
Living alone	**0.728 (0.585–0.905)**	**0.004**	**0.711 (0.570–0.887)**	**0.002**	**0.702 (0.547–0.901)**	**0.005**
Age (≥80 years)			**1.246 (1.048–1.483)**	**0.013**	**1.230 (1.013–1.494)**	**0.037**
Sex (female)			0.938 (0.789–1.116)	0.471	0.869 (0.693–1.091)	0.226
Uneducated					0.893 (0.727–1.098)	0.283
Marital status (SDWS)					1.096 (0.883–1.362)	0.405
Residence (Rural)					0.992 (0.826–1.191)	0.928
Financial support (insufficient)					1.026 (0.806–1.306)	0.835
Sleep quality (bad)					1.097 (0.798–1.506)	0.570
Sleep duration						
Short					0.719 (0.485–1.068)	0.102
Long					1.046 (0.824–1.328)	0.710
Medium					Ref.	
Smoking						
Current, n (%)					0.832 (0.641–1.079)	0.165
Ex-smoker, n (%)					0.873 (0.643–1.184)	0.383
Never, n (%)					Ref.	
Drinking						
Current					0.983 (0.767–1.261)	0.894
Ex-drinker					0.941 (0.682–1.298)	0.710
Never					Ref.	
Exercise					0.969 (0.799–1.177)	0.753
Physical labor					0.860 (0.673–1.099)	0.228
Depression					0.829 (0.621–1.106)	0.202
ADL disability					1.003 (0.713–1.412)	0.985
mMMSE					0.992 (0.970–1.015)	0.490
Hypertension					1.122 (0.918–1.372)	0.261
Diabetes					0.847 (0.608–1.179)	0.324
Heart disease					1.126 (0.852–1.487)	0.405
Stroke					**1.486 (1.085–2.035)**	**0.014**
Respiratory diseases					1.122 (0.840–1.498)	0.435
tuberculosis					0.607 (0.342–1.079)	0.089
Cancer					1.050 (0.527–2.093)	0.889
Parkinson’s disease					1.517 (0.710–3.241)	0.281
Arthritis					0.812 (0.588–1.121)	0.206

OR, odds ratio; CI, confidence interval; SDWS, separated/divorced/widowed/single; ADL, activities of daily living; mMMSE, modified Mini Mental state examination.

Bold values represent statistical difference.

Table 3 shows the results of subgroup analysis. LA was significantly associated with CC decline in 65–79 years old, female and city/town subgroups: the adjusted ORs of LA group were 0.619 (95% CI: 0.407–0.942, *p* = .025), 0.567 (95% CI: 0.408–0.790, *p* = .001) and 0.461 (95% CI: 0.310–0.685, *p* < .001) respectively. LA was not significantly associated with CC decline in advanced age (≥80 years old), male and rural subgroups.

**TABLE 3:** Associations between CC decline and LA by subgroup analysis.

	Crude model		Adjusted model	
	OR (95% CI)	*p*	OR (95% CI)	*p*
65–79 years old	**0.628 (0.443–0.891)**	**0.009**	**0.619 (0.407–0.942)**	**0.025**
≥80 years old	0.764 (0.575–1.016)	0.065	0.760 (0.553–1.043)	0.090
Female	**0.556 (0.412–0.750)**	**<0.001**	**0.567 (0.408–0.790)**	**0.001**
Male	1.034 (0.746–1.432)	0.841	0.960 (0.646–1.428)	0.841
Rural	0.875 (0.659–1.317)	0.354	0.943 (0.676–1.317)	0.731
City/town	**0.560 (0.396–0.794)**	**0.001**	**0.461 (0.310–0.685)**	**<0.001**

Adjusted model: adjustment for age, sex, education, marital status, residence, financial, sleep quality, sleep duration, smoking, drinking, exercise, physical labor, depression, ADL disability, mMMSE and comorbidities, the grouping variable were excluded in each subgroup analysis.

Bold values represent statistical difference.

## 4 Discussion

To the best of our knowledge, this is the first longitudinal study to evaluate the association of living arrangements with CC decline among older adults. Both univariate and multivariate logistic regression results of our study showed that LA was a protective factor for CC decline, which might help preserve skeletal muscle mass and reduce the risk of sarcopenia. The results of cross-sectional and longitudinal study about living arrangements and frailty were different (Woods et al., 2005; Yamanashi et al., 2015), and the result of our longitudinal study was also inconsistent with the result of cross-sectional study (Cheng et al., 2021). The association of LA with CC decline seems to be specific for older adults in 65–79 years old, female and city/town subgroups when conducting the subgroup analysis.

The reason why LA was protective was not clear yet. In our results, the age structure, gender ratio, education level, marital status, urban-rural distribution, economic status, sleep duration, smoking and drinking status, and mMMSE score were different between LA and NLA groups; older, women, uneducated, SDWS, rural, insufficient financial support, short-sleeper, non-smoker, non-drinker, and lower mMMSE scorer were more common in subjects living alone. These variables with statistical difference above, such as lower economic status and educational level in LA group, didn’t seem to be beneficial for skeletal muscle mass maintenance and sarcopenia prevention (Brennan-Olsen et al., 2020). In addition, LA was often accompanied by decreased family and social support and social isolation for older adults (Lou and Ng, 2012; Teerawichitchainan et al., 2015), and thus modified the associations of loneliness with adverse health outcomes in previous study (Wei et al., 2022). Maybe less tobacco use could be protective (Locquet et al., 2021). However, the results were sustained in multivariate logistic analyses adjusted for these covariates. There must be some other mediators that make LA protective for CC decline.

Gu et al. presented a theoretical framework of reciprocal causality between LA and health. That is, the solitary living arrangement needs to be conceptualized as the cause of subsequent health outcomes as well as the outcome of the prior health status (Gu et al., 2019). Health status of older adults affected their preference for living arrangements and both physical and mental health conditions play a role in the transitions of living arrangements (Brown et al., 2002). A disabled older adult who loses the capability to live independently would be more inclined to live with others. In addition, older adults living alone need to cope with more jobs of daily life by themselves, helping them get rid of their bad sedentary habits; and a better cognitive or physical function is needed to support them in handling the daily jobs. Physical activity, especially resistance exercise, which can improve skeletal muscle mass and strength, is the primary method for sarcopenia prevention and treatment (Cruz-Jentoft and Sayer, 2019). However, the BMI, exercise and physical labor, ADL disability, depression status, and comorbidities were all comparable at baseline and didn’t change the result in the adjusted model. And the mMMSE score was lower in the LA group probably due to lower education level. It suggests that the reciprocal causality alone cannot explain the findings.

LA does not always have adverse effects on physical and mental health, depending on the different context (Yeung and Cheung, 2015). Previous studies suggested that LA in older adults was not always a risk factor for health, and adverse health outcomes among older adults living alone might be confounded by poor social network (Sakurai et al., 2019). Women live longer than men and are more likely to be widowed. Compared to men, women tend to have larger social networks (Cornwell, 2011), which may explain the protective effect specific in women in our result. Another advantage of LA is that older adults who live alone can be relieved of family obligations and have more free time (Eshbaugh, 2008; Gu et al., 2019), which may offset the adverse effects of LA for physical and mental health. Actually, other study also found that women living alone have better psychologic function or lower frailty risk than women living with a spouse (Michael et al., 2001; Trevisan et al., 2016). Compared to very old adults over the age of 80 or rural residents, people aged 65 to 79 or urban residents in China have more energy or better conditions to do the things they love or to be socially active (Liu et al., 2019). This may explain the protective effect specific for older adults in 65–79 years old and city/town subgroups in our result. It reminds us that the public health policy should be specific for different area groups, different age groups, and different gender groups. The implementation of the newly proposed rural revitalization strategy of China will bridge the urban-rural gap and might reduce the risk of sarcopenia among rural older adults. And future research needs better quantification of social factors to differentiate between different context.

The strength of this study was that it was the first longitudinal study to investigate the relationship between living arrangements and CC decline, revealing the causality better. However, there were also limitations. First, the measurement of the CCs was not accurate enough, which were rounded up to the nearest integer in centimeters. And CC is recommended as one of the methods for sarcopenia screening in AWGS 2019 but not a gold standard for diagnosis. It could reflect but not substitute for skeletal muscle mass when it comes to sarcopenia. Second, we did not consider the change of living arrangements between two waves. The advantage, however, is that compared to other variables, such as sleep quality or sleep duration at different time, living arrangement is a more stable exposure in a 4-year time span (Nakakubo et al., 2021). Third, several potential confounders, such as social networks, were not included in our study. The better methods of social factors measurement are needed in future studies.

## 5 Conclusion

In summary, this study revealed that living alone was associated with a reduced risk of calf circumference decline among older adults, especially for older adults of 65–79 years old, women and city/town dwellers. It suggests that living alone may be a protective factor for skeletal muscle mass maintenance and sarcopenia prevention. Public health policy should be specific for different area groups, different age groups, and different gender groups. The results of this study will also provide a theoretical basis for the rural revitalization strategy of China to improve the health of rural older adults. Further studies are required to confirm these findings and identify the specific mechanisms that can be applied to improve the public health of older adults.

## Data Availability

The datasets presented in this study can be found in online repositories. The names of the repository/repositories and accession number(s) can be found below: https://opendata.pku.edu.cn/dataverse/CHADS.
